# The expansion of medical education in the Dutch East Indies and the formation of the Indonesian medical profession

**DOI:** 10.1017/mdh.2024.11

**Published:** 2024-04

**Authors:** Hans Pols

**Affiliations:** School of History and Philosophy of Science, The University of Sydney, Sydney, New South Wales, Australia

**Keywords:** medical education, Dutch East Indies, Indonesia, medical profession, public health

## Abstract

In 1851, the colonial administration of the Dutch East Indies established a two-year program to educate young Javanese men to become vaccinators in Batavia (today’s Jakarta). During the following sixty years, the medical curriculum was expanded several times; in 1913, it consisted of a ten-year program. In 1927, the Batavia Medical School, granting degrees equivalent to those of Dutch university-affiliated medical schools, commenced operations. Consequently, a steadily increasing number of Indonesian physicians with various credentials were employed by the colonial health service, plantations, sugar factories and mines, or established private practices. They became a social group that occupied an ambiguous and even paradoxical position somewhere between Europeans and the indigenous population. During the 1910s, this inspired these physicians to obtain credentials and professional recognition equal to those of their European colleagues. Several of them became active in journalism, politics and social movements. During the 1920s, several became radicalised and criticised the nature of colonial society. In the 1930s, following the increasingly repressive nature of colonial society, most of them remained active in the public sphere while a small group dedicated itself to improving medical research and health care. After the transfer of sovereignty from the Netherlands to Indonesia on 27 December 1949, this small cadre reestablished medical education and health care, and built the Indonesian medical profession.

In the Dutch East Indies – as elsewhere – medical education had profound effects on how aspiring physicians think, analyse, diagnose and address problems as well as their personality, emotional disposition, demeanour and, therefore, their personal and professional identities.^1^ A medical education also opened new and modern career opportunities and professional trajectories that had not previously been available to Indonesians. Indonesian physicians embraced new responsibilities, social and organisational roles, and professional identities by finding employment at plantations, mines, sugar factories and the colonial health service. Because of their educational attainments, sites of employment and personal preferences, several of them aimed to enter the European social sphere but encountered friction or even hostility when doing so. Given that the medical colleges in the Indies provided a practical and professional medical training, the degrees they granted were considered inferior to those awarded by European university-affiliated medical schools, which provided an academic training course. This led to tension and conflict between Indonesian physicians and their European colleagues, who viewed the former’s medical education and skills with disdain.

Indonesian physicians came to occupy an ambiguous and at times paradoxical social position between the colonisers and the colonised. Several of them therefore began to apply physiological and evolutionary metaphors in the analysis of political realities and advocated upgrading medical education in the colonies. Experiencing social and professional discrimination motivated social and political action among some of them, which ranged from founding a professional union and working as journalists to participating in the (rather limited) colonial political institutions. In 1927, the Batavia Medical School was established, offering degrees equivalent to those awarded by Dutch university-affiliated medical schools. Soon, small numbers of highly qualified Indonesian physicians graduated. Most of them were not politically active but dedicated themselves to medical research, treatment and education. This relatively small cadre of well-educated Indonesian physicians built the Indonesian medical profession, a medical infrastructure and medical schools after the transfer of sovereignty from the Netherlands to Indonesia in December 1949.

The historiography on these topics remains rather modest. The various institutions for medical education in the Dutch East Indies regularly published anniversary or memorial volumes, some of which provide excellent accounts.^2^ Preliminary work on the history of the Batavia medical college was undertaken by the physician and historian of medicine Daniël de Moulin.^3^ In 2011, Liesbeth Hesselink published her monograph *Healers on the Colonial Market: Native Doctors and Midwives in the Dutch East Indies*, which was the first one on the history of medical education in the Dutch East Indies. Hesselink’s study covers the period from 1850 to 1915 and includes both the (successful) Batavia medical college (STOVIA) and the (ultimately unsuccessful) school for Indonesian midwives.^4^ In 2018, my monograph *Nurturing Indonesia: Medicine and Decolonisation in the Dutch East Indies* appeared.^5^ It expanded on Hesselink’s study by extending the time period covered until after Indonesian independence, by including the Surabaya medical college (founded in 1913), and by focusing on the involvement of Indonesian physicians and medical students in the nationalist movement.

The current article draws on both monographs while further analysing the content of medical education, in particular the role of dissection, medical equipment and laboratory work; the organisation of medical education in the Netherlands and the Dutch East Indies; the role of public health; and the professional trajectory of several Indonesian physicians through the Pathological Laboratory in Medan, which had been established by the tobacco plantations in the Deli area on the East Coast of Sumatra. I start with analysing the nature of medical education in the Dutch East Indies from 1851 to 1942 and the ensuing formation of a corps of Indonesian physicians, their social and cultural struggles, and their professional and political engagement. I continue with an exploration of changes in medical education during the Japanese occupation, when several Indonesian physicians came to occupy leading positions in medical education, medical care and health services. They aimed to decolonise and Indonesianise medicine. After the transfer of sovereignty in 1949, these leading physicians faced the daunting task of building institutions for medical education, health care and public health.

## A modest start

In 1847, an epidemic (most likely typhoid fever) raged in the Banyumas area of central Java. The colonial administration asked Willem Bosch, the head of the colonial (military) medical service, to propose measures that could be undertaken to alleviate its effects.^6^ In a response his superiors found much too elaborate and critical, Bosch proposed several courses of action, one of which was establishing a two-year training course for indigenous young men aged 16 or over to become vaccinators and assistant physicians.^7^ In 1851, this course commenced in a few rooms of Batavia’s military hospital under the direction of medical officer and ichthyologist Pieter Bleeker. It was modelled on the military medical college in Utrecht (The Netherlands), which prepared future medical officers for their employment in the colonies.^8^ On the grounds of the Batavia military hospital, both a dormitory and classrooms were established. The training course was conducted in a boarding school and was generally referred to as the *Dokter Djawa* school (Javanese Doctors’ School; *Sekolah Dokter Djawa*); the school kept this name even though, a few years later, students of all ethnic backgrounds were allowed to enrol.^9^ It granted the degree of *Dokter Djawa* [Javanese physician]. In 1851, twelve students (named *élèves*) entered the course.^10^ Teaching was conducted in Malay, the *lingua franca* of the archipelago – the language of inter-island trade, markets and ports, known to some extent to most inhabitants of the archipelago, where over 250 languages were (and are) spoken.

Expectations were high even though the means were limited and the course of study exceedingly short. During the following decades, the duration of the medical course was regularly increased, and the curriculum expanded to better prepare students for their future vocation. In the reorganisation of 1875, training was extended to five years, consisting of a two-year preparatory course and a three-year medical course. That same year, Dutch became the medium of instruction. The school’s instructors increasingly felt that Malay was unsuitable, as they did not speak it well while for most students it was their second or third language. Moreover, the instructors struggled to formulate Malay equivalents for scientific and medical terms such as the elements of the periodic table and the various organs of the human body. Most of them merely avoided such linguistic challenges by focusing on teaching practical skills rather than anatomy, physiology, chemistry and physics in the hope of ‘training useful assistants instead of useless physicians’.^11^ After 1875, Dutch was taught during the preparatory course; it became compulsory to speak the language even during informal exchanges.

**Image 1.**
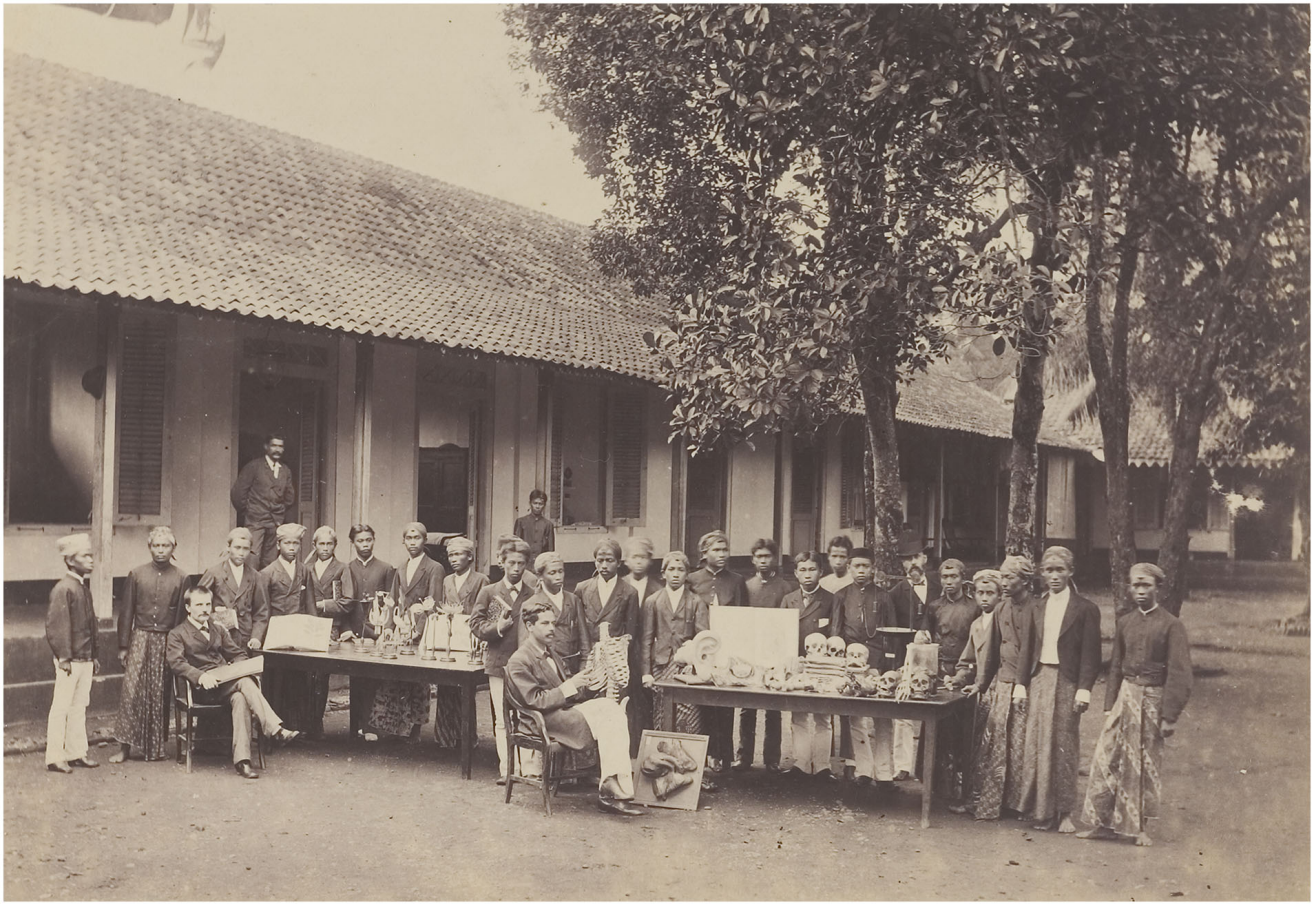
Students and instructors of the *Dokter Djawa* school in 1880. This image gives a good indication of the teaching material that was used in the medical course of study, which included several skulls and bones, an incomplete skeleton, models of organs (such as the large model of the human ear) and illustrations. Image from *Dokters-Djawa School, Batavia*, 1880. Image made available by Leiden University, KITLV 5185.

Despite the energetic efforts of the instructors at the *Dokter Djawa* school, results remained disappointing. A critic claimed that these instructors continued to ‘domesticate and tame [*dresseert*; lit. dressage] natives to perform as *dokter djawas*’.^12^ This critic argued that, because of the limited intelligence of the ‘natives’, the medical college merely trained indigenous young men to perform a few practical tasks which did not require any comprehension of medical science. The instructors at the *Dokter Djawa* school were medical officers with many other responsibilities; they were unable to spend much time on instruction. In addition, they were transferred frequently, which led to regular changes in teaching personnel and approaches to medical education. There were other reasons that conditions at the school were less than ideal. Teaching equipment was scarce. Instruction in anatomy, for example, was perfunctory and relied on large anatomical illustrations, a few paper maché models and some human bones (Image 1). In 1871, a request was submitted to acquire ‘a skeleton, various loose bones, [and] a cracked skull’.^13^ In 1877, a retiring medical officer sold a skeleton to the school. It was equipped ‘with copper hinges and springs, so that all normal movements can be executed’. In addition, ‘the origin and attachment of all muscles are indicated by colours and printed instructions’.^14^

Instructors often complained that the number of corpses available for instruction in anatomy and dissection was exceedingly low; because of the tropical climate, corpses decomposed rapidly and could only be used for up to two days. When a corpse became available in the military hospital, all other lessons were suspended to give the pupils as much time as possible to observe their instructor dissect the corpse. Students consequently only developed dissection skills to a minimal extent.^15^ The origins of these corpses were varied: some were referred for dissection by the courts, others came from the military hospital or the native hospital closer to the harbour, while the physical remains of forced labourers constituted a third source.^16^ Very few Europeans and other inhabitants of Batavia were willing to allow the corpses of deceased family members to be dissected, which contributed to the shortage of teaching material. The limited shelf life of available corpses made this bad situation even worse.

## Improvements in medical education

Conditions at the *Dokter Djawa* school improved significantly after 1890. Starting that year, only indigenous young men who had graduated from the European elementary school were accepted as students – generally at the age of 12 to 14. These elementary schools were primarily intended for the education of Dutch children; indigenous children from Dutch-speaking families (such as civil servants and the higher ranks of the indigenous aristocracy) were accepted in exceptional circumstances. Because the graduates of these schools had a good mastery of the Dutch language, medical students spoke Dutch with each other, which also reflected the social aspirations of their parents. The increasing ethnic diversity of the student body had made speaking in their native tongues practically untenable as well. As most members of the indigenous elite, students revered Dutch as the ‘avenue to the West’, and the language of science, modernity and progress, while the various languages of the archipelago came to represent outdated tradition at worst and the innocence of childhood at best.^17^ As one student opined in 1918: ‘Dutch is a perfect language … the key to the temple of knowledge and the arts … able to bring us to our goal of the highest level of development’.^18^ From the turn of the twentieth century, the persistent linguistic problems that had plagued medical education vanished.

A second factor that improved medical education was the arrival, in 1888, of Christiaan Eijkman.^19^ Eijkman, a leading medical researcher in the Netherlands, had been sent to the Indies on a special mission to investigate the cause of beriberi, which continued to claim vast numbers of casualties in prisons, plantations and boarding schools. He was appointed as the director of the Medical Laboratory at the Batavia Military Hospital and of the *Dokter Djawa* school, which meant that both positions were now occupied by a civilian with a full-time appointment rather than by a regularly changing cast of medical officers. Inspired by what has been called the bacteriological revolution, Eijkman expected to find the bacterium responsible for beriberi; instead, he discovered that it was the result of a nutritional deficiency. His work later inspired medical research on the roles of vitamins; in 1929, he received the Nobel Prize for this research.^20^ Because of the importance of his mission, Eijkman had been supplied with the latest and most advanced medical equipment to conduct bacteriological and anatomical research from the Netherlands—including microscopes, everything needed to make anatomical preparations for microscopic analysis and instruments to perform dissections.^21^ Eijkman’s presence had a great effect on the morale at the school as it was now headed by a medical researcher of international renown. In addition, he allowed all medical students access to the Medical Laboratory after hours, which meant that they were trained in using the most advanced equipment available at the time.

In 1896, Eijkman returned to the Netherlands. He was succeeded by H. F. Roll, who proposed to expand medical training to six years. In the meantime, what has been named the parasitological revolution in tropical medicine was taking place and X-ray equipment had been introduced in the Indies. Both greatly enhanced the reputation of modern Western medicine and held the promise that effective therapeutic interventions would be available in the near future.^22^ In 1899, a leading and exceedingly wealthy plantation owner in Deli (the area around Medan on the east coast of Sumatra) had instituted an expansive public health program which promised to reduce the loss of manpower through disease and death.^23^ The same year, he and two other wealthy Deli plantation owners donated funds for the erection of a new school building. The new building was completed in 1902, and the following year, Roll’s proposed expansion of the school’s curriculum was implemented. The institution was renamed School for the Education of Native Physicians (*School tot Opleiding van Inlandsche Artsen*; STOVIA); it granted the degree of native physician (*Inlandsch Arts*). The new building had ample room for teaching, laboratory experiments and microscopy. It also had a dormitory housing all students.

**Image 2.**
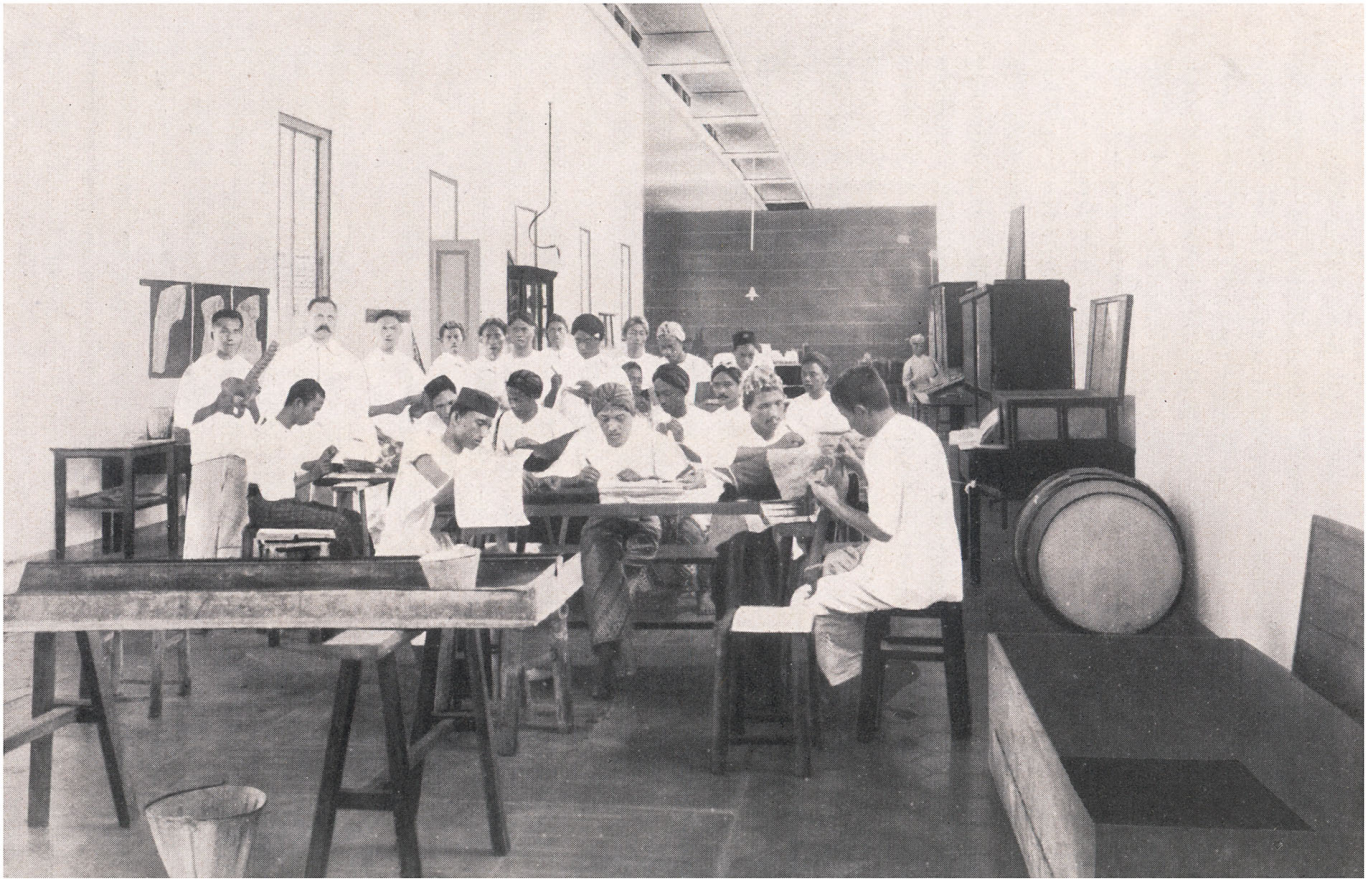
Instruction in anatomy at the STOVIA in 1908. Standing, second from the left, is instructor T. G. van Vogelpoel. From A. de Waart (ed.), *Ontwikkeling van het Geneeskundig Onderwijs te Weltevreden, 1851–1926* (Weltevreden: Kolff, 1926), 154.

In addition to the use of the Dutch language and the initiatives of Eijkman and Roll, the introduction of new techniques for the long-term preservation of human cadavers was a third factor resulting in the improvement of medical education. In the 1880s, physicians in Europe started to experiment with formaldehyde and formalin for this purpose.^24^ In 1904, T. G. van Vogelpoel, instructor in anatomy, physiology and histology, used formalin through arterial injection and placed corpses thus prepared in a bath of diluted formalin.^25^ Through this method, corpses could be preserved for months or even years. Unfortunately, the new building did not have facilities for anatomical demonstrations; an unused dormitory room was therefore transformed into an anatomical theatre (Image 2). Within a few years, a dedicated room for instruction in anatomy became available.

In 1901, Queen Wilhelmina of the Netherlands and the Dutch Empire announced an important change in colonial governance on behalf of the Dutch government. From then on, it would assume the ethical responsibility for the welfare of its colonial subjects. While previous colonial activity was driven by extracting profits for the Dutch Treasury and Dutch private initiative, colonial policy in the twentieth century would instead become a civilising mission to bring progress and prosperity to the Indies. The Ethical Policy aimed to realise the development of country and population population through education, irrigation and transmigration, and constituted a fourth factor benefiting medical education in the Indies.^26^ In 1911, the Civil Medical Service [*Burgelijke Geneeskundige Dienst*] was established separately from the Military Medical Service – belatedly, as physicians had been advocating this separation for decades. In the spirit of the Ethical Policy, the colonial administration finally accepted a (somewhat limited) responsibility for the health and medical care of the indigenous population. From the 1870s on, plantations, businesses and missionary associations had begun to provide medical services to the indigenous population – the colonial state was a latecomer in that respect. According to several physicians and colonial administrators, medical care would best be provided by Indonesian physicians, as they were trusted by the population and understood their fears and concerns about disease and Western medicine.

In 1913, the medical course was extended again, to seven years; the preparatory training remained three years long, making the total course of study ten years. At that time, the Batavia medical college also started accepting Chinese Indonesian and Indo-European students; their number remained small, as their families preferred to send their sons (and a few daughters) to medical schools in the Netherlands. Both medical colleges began accepting female students. The name of the Batavia college was changed to School for the Education of Indies Physicians (*School tot Opleiding van Indische Artsen*; the acronym remained STOVIA); the degree granted became Indies physician (*Indisch Arts*). The same year, the Netherlands Indies Doctors’ School [*Nederlandsch-Indische Artsen School*; NIAS) was established in Surabaya, following the same course of study and granting the same degree. A steady stream of graduates filled the lower ranks of the Civil Medical Service (in 1925, it was renamed the Public Health Service (*Dienst Volksgezondheid*; DVG)), entered private practice or were employed by plantations, mines and other businesses. Indies physicians could soon be found in most medical services in the Indies.

**Image 3.**
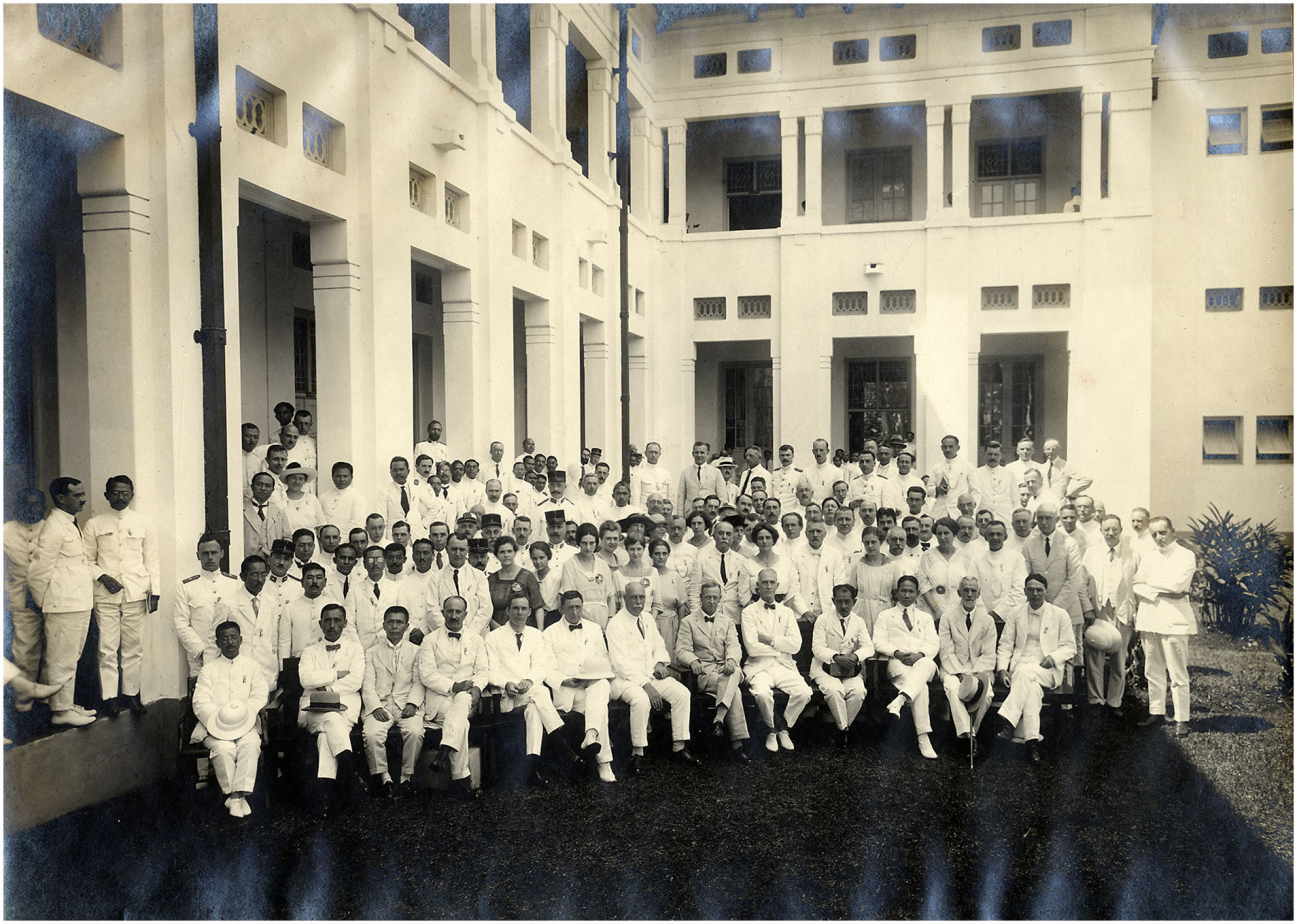
The fourth Congress of the Far Eastern Association of Tropical Medicine, held in August 1921 at the new premises of the STOVIA, indicating the pride leading physicians and the colonial administration took in the new building. From 1927 on, these premises housed the Batavia Medical School (*Geneeskundige Hoogeschool*). Image made available through the Leiden University library, KITLV 68926.

**Image 4.**
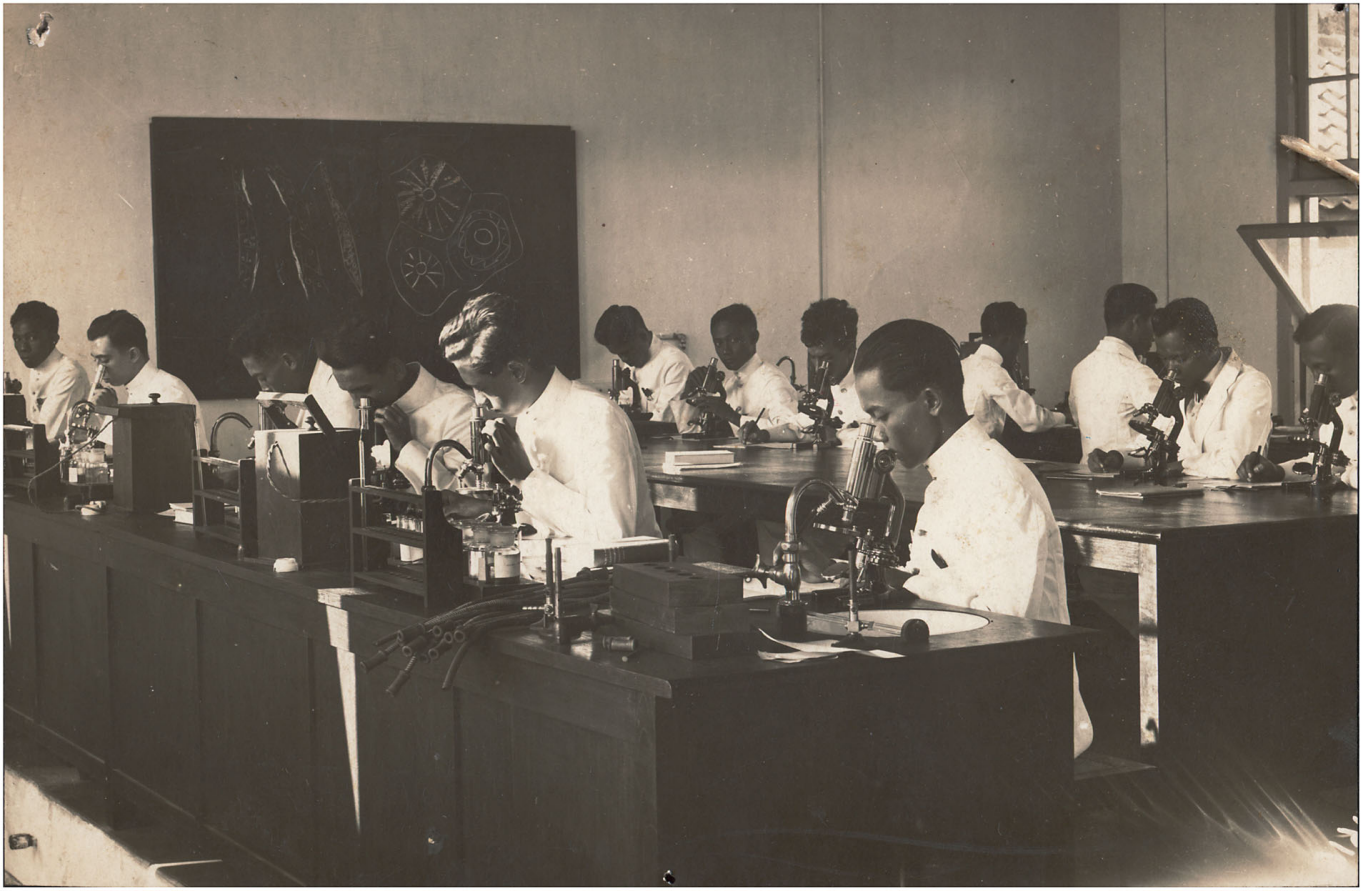
Practical instruction in microscopy at the STOVIA in 1924 when it had relocated to the new medical school building. Image: author’s collection.

A last improvement in medical education took place in 1919, when the STOVIA moved to a new building (Image 3), which was part of the new medical precinct at Salemba, where the Central Civilian Hospital and the Medical Laboratory (later named the Eijkman Institute) were located (the new medical school building currently houses the Faculty of Medicine at the University of Indonesia). Officers of the Rockefeller Foundation had regularly visited the Dutch East Indies to survey medical education, the health service and public health initiatives.^27^ As Hohee Cho shows in this issue, the Rockefeller Foundation was also active in several Pacific islands around the same years, becoming a major nongovernmental actor in the health sector throughout the region. In 1916, Victor Heiser wrote about the STOVIA that ‘everything considered, this is a very good school’.^28^ He recommended providing financial support in the near future. This support was not forthcoming because the Foundation ‘did not give assistance to the lower type of medical education’ – that is, the medical colleges like those in the Indies.^29^ In 1924, after the STOVIA had relocated, Rockefeller officer W. S. Carter reported: ‘The STOVIA medical school has a splendid physical plant and the arrangement of unifying three institutions (the medical school, civil hospital and government laboratory) practically into one by the closest cooperation between them, is ideal’.^30^ Nonetheless, he thought that although the laboratories were well equipped, teaching remained mostly didactic in nature – too many hours were devoted to lectures and there was ‘too little practical laboratory teaching’ (Image 4).^31^ According to him, there was not enough laboratory equipment available to make more laboratory-based teaching feasible. He was nonetheless impressed with the new teaching facilities.

## Medical students: embracing modernity

After the turn of the twentieth century, students who entered the medical colleges in the Indies received a modest bursary for expenses such as books, food and incidentals.^32^ In exchange, they were obliged to work for the colonial medical service for a period of ten years for relatively modest wages. This type of financial arrangement to channel medical graduates into specific roles in the colonial medical services appears to have been relatively common, with examples found in India as early as the beginning of the nineteenth century, as Martin Robert reports in this issue. Consequently, the STOVIA (and, from 1913 on, the NIAS) attracted applicants from the lower ranks of the indigenous aristocracy. These applicants met the entry requirements, but their families could not afford to pay tuition fees or living allowances. They had attended a Dutch primary school, and most had received some additional education.^33^ The only way to secure their future social status was to pursue additional educational opportunities. The Indies medical colleges were therefore widely seen as schools for the impecunious and poor; they were often referred to as the *sekolah miskin* or poor schools.^34^ Students came from relatively poor households as compared to the higher aristocracy, representatives of which served as regents and high colonial administrators. Compared to farmers, fishermen, craftsmen and domestic servants, they were still very wealthy.

In establishing a colonial state, the Dutch colonial administration had co-opted the upper ranks of the higher aristocracy by appointing them as regents [*bupati*] or to other high administrative positions while providing them with generous allowances. They were consequently able to safeguard the future career of their sons through their wealth and connections. Indigenous schools for future higher administrators – nicknamed *sekolah raja* [royal schools] – offered a course of study lasting two years with high tuition fees, while the salaries offered to its graduates far exceeded what any Indies physician could ever expect to earn. The marked differences in social background, social status and wealth of the lower and higher indigenous aristocracy caused tension and friction between them. Medical students often depicted regents and their families as relying on family connections, wealth and the exploitation of farmers rather than on knowledge and skills; they lived undeserved opulent lives seemingly devoted to traditional arts and culture while maintaining strict social hierarchies. Medical students and Indonesian physicians instead emphasised their training, skills and knowledge to argue for improvements in their social position. They strongly believed that they were contributing to the health and welfare of the indigenous population. They embraced Western science, medicine and technology as means to improve conditions for the archipelago’s population and welcomed the promises of progress [*kemajuan*] and modernity. Cosmopolitanism, an identification with science and medicine and with the medical profession allowed these physicians to craft a new class identity.^35^ Indonesian physicians constituted a professional upper middle class within indigenous society and, thereby, a new elite – a colonial bourgeoisie that distinguished itself on the basis of its social capital, consisting of education, degrees, skills and professional status, rather than on the basis of capital, inherited privilege or ethnicity.

Many Indonesians who had received some Western-style education found employment in the lower echelons of Dutch businesses and colonial institutions. They had started to discuss ideas on progress and modernity in the budding vernacular press in the 1880s.^36^ In the late 1890s, progressive Dutch journalists expressed similar ideas as well, arguing for a reorientation of colonial policy that was later endorsed by the Dutch government when it proclaimed the Ethical Policy. After the turn of the twentieth century, Indonesian physicians and medical students participated in these discussions, giving them distinct inflections. They could be named as ‘conscripts of modernity’, using the phrase introduced by David Scott, as well as adherents to cosmopolitanism – an imagined and idealised world where education and accomplishment determined one’s status rather than race, ethnicity or nationality.^37^ They eagerly adopted Western (Dutch) habits, culinary preferences, social styles and appearances. Medical students expressed their ideas and ideals in student newspapers, school almanacs and in the general press.^38^

On 20 May 1908, a group of medical students founded *Budi Utomo* (Beautiful Endeavour or Prime Philosophy), an association of Javanese students advocating expanded (Western-style) educational opportunities for Javanese youngsters. It sought to unite all Javanese students in social activities and sporting events. Despite its lack of revolutionary zeal and any reference to independence as well as its enthusiastic support of Dutch educational initiatives, the founding of *Budi Utomo* is now seen as the origin of the Indonesian nationalist movement.^39^ Because the ideas it propounded resembled those of the advocates of the Ethical Policy, it was embraced by Dutch progressive social commentators as the start of the awakening of the Indies.^40^ Since 1948, *Budi Utomo’s* founding is commemorated every year on National Awakening Day (*hari kebangkitan nasional*; 20 May). The premises of the Batavia medical college (STOVIA) now houses the Museum of National Awakening (*Museum Kebangkitan Nasional*). Irrespective of whether *Budi Utomo* was nationalist and revolutionary, its founding indicated the social and political engagement of Indonesian medical students.

In the late 1910s, medical students established and headed various youth associations, including *Jong Java* [Young Java], the *Jong Sumatranen Bond* [Association of Young Sumatrans], *Jong Minahasa* [Young Minahasa] and several others. Even though these associations officially focused on traditional culture and sports, they also became places where students debated colonial politics. More importantly, the activities of these groups brought students together for the first time. This bolstered their identification, initially, with their ethnic groups and, subsequently, with all young Indonesians, a new and positive identity that all inhabitants of the archipelago shared as subjects of the Dutch colonial empire in Asia. Through frequent interactions and activities, they came to see themselves as part of an imagined community of Indonesians, a community they later came to realise through political and literary activities, and, ultimately, through the war of independence.^41^

## Indonesian physicians: emancipation and nationalism

In the Netherlands, a variety of secondary educational institutions were established during the second part of the nineteenth century. Education was (and is) provided in three tiers, corresponding to subjects taught and the expected career paths of its graduates. The highest level of education was provided by the Higher Civil School [*Hoogere Burgerschool*; HBS], which prepared its pupils for the most respectable professions and university entry with a course of study lasting five to six years.^42^ The HBS primarily catered to the children of the elite. The second tier consisted of schools extending the primary school curriculum (More Comprehensive Lower Education (*Meer Uitgebreid Lager Onderwijs*; MULO)) and provided training for typical middle-class professions like accounting, lower management, shopkeeping and, later, auxiliary health personnel. It provided an avenue for social advancement for the ‘common people’, for whom the HBS was out of reach.^43^ The lowest level of education was provided by occupational and trade schools [*ambachtsscholen*], which offered practical training over a period of two to four years for future electricians, carpenters, house painters and the like.

There were only a few HBS schools in the Indies, and they primarily catered to the children of the Dutch elite, although a very small number of Indonesian children attended as well. The MULO became very popular in the colonies because of its emancipatory function. Because the two medical colleges in the Indies provided a practical or professional education, and because teaching was highly structured through regular exams and supervised study, the education these colleges provided was considered practical rather than academic in nature – more akin to the training provided to auxiliary health personnel than academic physicians. The salary scales for its graduates reflected this (as salary scales were based on degrees attained).^44^ Indies physicians earned far less than their European colleagues because they held qualifications that were considered of lower quality. The difference in the medical qualifications held by Indies and European physicians, and hence the secondary position of the former in the colonial medical service caused resentment, friction and frustration, in particular because European physicians continuously emphasised (and exaggerated) the vast differences in training both groups had received. It consequently inspired initiatives among Indies physicians to improve their qualifications, demand better working conditions and gain access to Dutch medical schools. In the 1920s, their activism became more political in nature, when several leading physicians began to denounce the ‘dualism’ in colonial medicine, as it was based was on the racial and ethnic distinctions inherent to colonial society. It was not uncommon in colonial societies to appoint indigenous physicians in subordinate ranks, which was justified by the inferior nature of the medical degrees obtained in the colonies. In the 1920s, Indonesian physicians started to interpret this state of affairs as an example of the imperial nature of colonial society.

Until 1924, when a technical college was established in Bandung, the medical colleges provided the highest form of education available in the Indies – and the only one that included training in scientific theories and methods. Despite that, their graduates were considered second-rate physicians and, often, as unwelcome interlopers in the European social sphere. Even worse, they were often treated as ‘mere natives’ by Europeans. They came to occupy contested and often untenable social positions; in Ann Laura Stoler’s words, they ‘ambiguously straddled, crossed, and threatened … imperial divides’.^45^ Indies physicians consequently became strongly aware of the rules and regulations that codified the ‘medical dualism’ (the differential treatment of European and Indies physicians) in the Indies and engaged in activities to improve their professional position. In the 1920s, they started to analyse the social conditions of colonial society as they came to interpret their inferior social and professional position as based on the nature of colonial society.

There were several ways in which Indies physicians could improve their professional status. Several of them went to the Netherlands to receive additional medical instruction and a medical degree from a Dutch medical school. Some were able to finance their studies in the Netherlands themselves; others relied on bursaries provided by the colonial administration. Those Indies physicians who had attained Dutch medical degrees played key roles in initiatives to improve professional conditions among Indies physicians. In 1912, several leading Indonesian physicians founded a union for Indies physicians: the Association of Indies Physicians (*Vereeniging van Indische Artsen*; VIG) with the aim of improving their professional position. Initially, it submitted polite requests to the colonial administration, with minimal results.^46^ In 1918, it became politicised and threatened to organise a physicians’ strike if their demands for increased remuneration were not met (at the last moment, the colonial administration gave in).^47^ In the 1930s, it started to advocate alternative plans for the organisation of medical care, modelled upon the demonstration projects of the Rockefeller Foundation in rural health.^48^ Dutch physicians and officials within the colonial administration were suspicious of these American health initiatives, as the United States was a foreign imperial power while the Rockefeller Foundation had been funded by the profits of Standard Oil; its associated entities were competing to access the rich oil resources in the Indies. Indies physicians, on the contrary, leveraged the influence of this alternate imperial medical power to enhance their own status and cultural capital.

Because of their strong beliefs in progress, development and modernity, and their outrage about being seen and paid as second-class physicians, several Indonesian physicians became public intellectuals by writing for and publishing newspapers, establishing and participating in associations, labour unions and, later, political parties. A number of them were elected to city councils and the colonial parliament (the *Volksraad* (Popular Council), established in 1918), where they advocated public health measures, more generous funding for health care, and improvements in educational standards. In 1902, Abdul Rivai, who had graduated from the Batavia medical college in 1898, introduced *Bintang Hindia* (Star of the Indies), an illustrated magazine featuring articles on everyday life in the Netherlands, the importance of education and the benefits of modern civilisation.^49^ At that time, he was studying medicine at the University of Amsterdam to attain a Dutch medical degree. In 1918, he became a member of the colonial parliament. Until the Japanese occupation of the archipelago in 1942, the *Volksraad* always counted at least one Indonesian physician amongst its members; at one time, there were three. They frequently brought up medicine, health and medical care, and repeatedly argued for improvements in the professional status of Indies physicians. The colonial parliament merely had an advisory role; its recommendations were hardly ever implemented by the colonial administration of the Dutch East Indies and the Dutch parliament.

Before Indies physicians started to engage in professional and colonial politics, medical education and the status of Indies physicians had were hot topics in discussions about colonial governance. Opponents of the Ethical Policy were more outspoken during the 1910s and became mainstream in the 1920s and 1930s. They were critical of attempts to offer a medical education (or any form of higher education) to Indonesians, arguing that ‘native’ brains were not able to benefit from it. According to them, higher education would only create an underclass of disgruntled urban-based pseudo-intellectuals alienated from their ethnic group and eager to foment political unrest.^50^ Their critique initially focused on medical education. In 1908, a report on the reorganisation of the colonial Medical Service appeared, arguing that the STOVIA’s curriculum needed to be curtailed, simplified and abbreviated in order to graduate larger numbers of much-needed Indies physicians.^51^ The committee argued that the Indies physician should be a ‘cheerful, skilled practitioner able to treat the most common forms of disease and injury’. He should therefore be a practical physician rather than a theorist: ‘half-baked scientific knowledge would be fatal for our native physicians, [who should] not be encumbered by half understood or misunderstood scientific theories’.^52^ Indies physicians only needed to be able to treat the most common tropical diseases under the supervision of their European colleagues. The report argued that the Indies needed a larger number of physicians; the Batavia medical college could only expect to meet this demand by increasing the number of its graduates, which meant shortening and simplifying the training it provided.

H. F. Roll, formerly director of the STOVIA, immediately penned a fierce defence of the institution he had built and expanded.^53^ Maluku physician W. K. Tehupeiory, at the time studying medicine at the University of Amsterdam and, later, founder of the Association of Indies Physicians, defended the STOVIA as the first Indonesian ever addressing the august *Indies Society* in The Hague.^54^ Several Indies physicians then studying in the Netherlands published a booklet arguing for further improvements in medical education in the Indies, which was the first time they had expressed their opinion collectively.^55^ Most Indies physicians found the *Report* deeply offensive; it provided an important motive for organisational activity among them. In 1913, when the Surabaya Medical College was established, the Union of (European) Physicians published a highly offensive and racist pamphlet opposing the medical education of Indonesians because it was a ‘mockery and revilement of the excellent education offered by our Dutch universities’.^56^ When the position of Indies physicians and the Batavia medical college became politicised in debates on the nature and the desirability of the Ethical Policy, it became an urgent matter for Indonesian physicians to organise themselves and speak with a collective voice.

## Further improvements

**Image 5.**
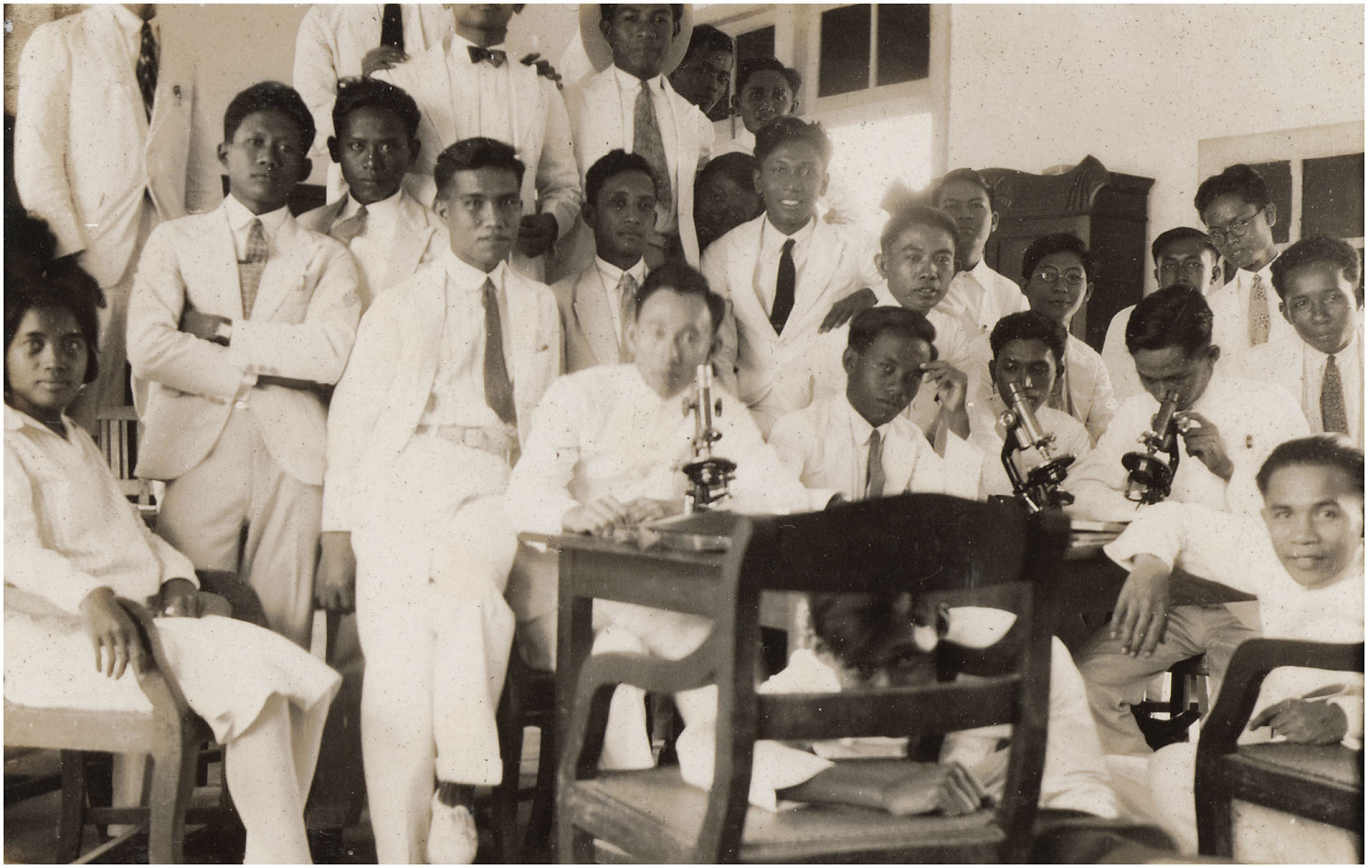
Humorous student photograph taken at the NIAS in the 1930s. Notice the female student on the left. Photo album of Soenarjo, NIAS graduate, author’s collection.

The members of the Association of Indies Physicians explored several avenues to reduce the ‘dualism’ in medicine in the Indies. They were deeply dissatisfied with their low wages and their secondary position within the colonial medical service. A small number of them opted to attain a medical degree at a Dutch university-based medical school. Abdul Rivai was the first one to migrate to the Netherlands to do so; about a dozen others followed before World War I. They advocated for bursaries to make advanced medical education in the Netherlands feasible; a modest number of bursaries were granted to this end during the next three decades. A second strategy to attain this result was the establishment of a medical school in the Indies that would grant degrees equivalent to those in the Netherlands. In the absence of a university, a School for Higher Medical Education [*Geneeskundige Hoogeschool*; GH; hereafter Batavia Medical School] enrolled students from 1927, granting degrees equivalent to those of Dutch medical schools.^57^ At the same time, the STOVIA no longer admitted new students and planned to close when all students had graduated; current students were encouraged to transfer to the Batavia Medical School. The NIAS continued to operate without any modifications (Image 5). Unlike the STOVIA and the NIAS, which only accepted students from the Indies, the Batavia Medical School accepted students from all ethnic backgrounds.

Facilities at the Batavia Medical School were impressive. As mentioned before, it was located next to the Central Civilian Hospital, which served as an academic hospital. The Medical Laboratory was located in close proximity, and the Institute for Public Health and Hygiene was established nearby in 1933. Facilities were comparable to those at the best European medical schools. Education at the Batavia Medical School was based on the ‘free study’ academic model common at Dutch (and German) medical schools and was primarily oriented towards fostering research skills. Students studied at their own pace and examinations were held only once a year. Consequently, only a small number of highly qualified medical researchers graduated each year. The Batavia Medical School therefore did very little to graduate a large number of much-needed physicians.

Once the Batavia Medical School started accepting students in 1927, one of the main obstacles for requesting funding from the Rockefeller Foundation had disappeared. Its professors, and in particular its successive presidents, met with representatives of the Foundation, at times submitting requests for funding. Most of these requests were denied. In 1934, Professor Cornelis Bonne, then president of the Faculty of Medicine, complained that his school had never received financial support.^58^ He had been unsuccessful in requesting funds for a new building to combine four separate medical libraries then operating in Batavia. A later funding request, for a new building for the Department of Pharmacy, was also declined.^59^ Several professors hoped to observe the organisation of medical education in the United States and to gain additional experience in their fields of specialisation. A number of them received fellowships, which were also granted to a number of Indonesian physicians, among them Sardjito, Djamil, Arifin and Raden Mochtar.

The officers of the International Health Board of the Rockefeller Foundation preferred to provide funding for public health projects, which focused on the health of populations instead of curative interventions or laboratory research. Several of the Board’s officers attempted to interest the teaching staff of the Batavia Medical School in public health, but the latter were mostly interested in clinical matters. One observer noted that ‘none of the Dutch in Java have any idea of modern teaching of public health or how to go about the same’.^60^ One exception was Eduard Willem Walch, who had worked at a plantation hospital in the Deli area and had received training in public health at Johns Hopkins University. In 1929, he was appointed as professor of hygiene and bacteriology.^61^ In 1933, the Institute of Hygiene and Bacteriology was established in close proximity to the medical school, funded by the Queen Wilhelmina Jubilee fund and hence called the Queen Wilhelmina Institute.^62^ It appeared that public health was now taken seriously at the Batavia Medical School.

The Institute of Hygiene and Bacteriology supported the rural hygiene demonstration projects funded by the Rockefeller Foundation, which were led by John Hydrick in the Banyumas area.^63^ It initiated a public health project in the Tanah Tinggi neighbourhood adjoining the medical school, aiming to stimulate research in public health and provide practical experience for medical students. It received financial support from the Foundation. Unfortunately, Walch died in December 1934 as a passenger on the highly publicised flight by KLM plane the *Uijver*, which was delivering Christmas mail from the Netherlands to Batavia but crashed in the Syrian desert leaving no survivors.^64^ Walch’s untimely death halted the further development of public health education at the Batavia Medical School. J. E. Dinger was appointed as Walch’s successor in July 1935, but the fomer’s main interests lay in laboratory work and not in public health. He did, however, continue to guide the public health project in the Tanah Tinggi neighbourhood, which continued after Indonesia’s independence.

The activities of several wealthy plantation owners in the Deli area around Medan, East Sumatra, had important ramifications for medical research and career advancement for Indonesian physicians. The public health activities in the area and the financial support for a new building for the Batavia medical college have been mentioned above. To support local public health initiatives, the Deli Planters Association established the Medan Pathological Laboratory in 1906 (it was later donated to the Civil Medical Service).^65^ Its main functions were to perform diagnostic tests for the various plantation hospitals in the area and to conduct medical research. In 1899, the head of the public health program at the Senembah and Deli plantations, the German physician W. A. P. Schüffner, had developed a diagnostic test for malaria through stained microscopic slides containing blood. The Pathological Laboratory became renowned for both its public health and its malaria research. Several medical researchers it employed later embarked on academic careers in the Netherlands. Schüffner, for example, became director of the Institute of Tropical Hygiene at the Royal Institute of the Tropics and professor in tropical hygiene at the University of Amsterdam.^66^ Several Indonesian physicians who worked at the Medan Pathological Laboratory were subsequently associated with the same institute to conduct research for a medical doctorate.^67^

The Civil Medical Service had undertaken and supported various public health initiatives as well. It was led by the architect of the Public Health Service, W. T. de Vogel. He transformed the Civilian Medical Service, which focused on providing treatment facilities, into a service that emphasised public health initiatives, leaving hospitals and clinics to private initiative.^68^ As city physician of Semarang (1901–1911) and member of the city council (1906–1911), he had ample experience in public health measures as he had advocated and overseen the establishment of sewage and fresh drinking water supply systems. He had also recommended that the city should be extended to the south, a mountainous area, instead of near the ocean where malaria was rife (the new area, *Candi Baru*, was indeed established to the south). De Vogel had identified the anopheles responsible for transmitting malaria in the area near the ocean – which he named *Ludlowi A. Rossi* – the first such one to be discovered that preferred to live in brackish water.^69^ De Vogel maintained close ties with Schüffner and initiated several projects to eradicate malaria through ecological interventions.^70^ Yet despite strengths in public health research and public health interventions in the Deli area and the extensive implementation of public health measures by the colonial Public Health Service, public health continued to remain a negligible component in the curriculum at the Batavia Medical School. Research in public health was outstanding at the Pathological Laboratory in Medan and, later, at the Institute of Tropical Hygiene at the Royal Tropical Institute in Amsterdam. At the Batavia Medical School, it remained marginal.

Contrary to the expectations of most Indies physicians, the Batavia Medical School did not improve the professional status of Indonesian physicians. Because of adverse economic conditions, the colonial administration had discontinued bursaries for medical students. Consequently, Dutch, Indo-European and Chinese-Indonesian students predominated; only a limited number of Indonesian students could afford to enrol.^71^ Nevertheless, the small but growing number of Indonesian physicians with academic medical degrees became an elite group of highly educated physicians within the Indonesian medical community. Unlike their colleagues with degrees from the Indies medical colleges, most of them did not participate in social and political activities; instead they focused on creating a cadre of well-educated Indonesian physicians which, eventually, could lead hospitals, the public health service, research institutions and medical schools.^72^ Under their stewardship, the journal of the Association of Indies Physicians, which had previously been devoted to issues related to their professional position, now concentrated on publishing medical research to demonstrate that their acumen in conducting research rivalled that of their Dutch colleagues.^73^ This small elite took control of public health, medical services, medical education and medical research during the Japanese occupation (1942–1945) and after Indonesia’s independence.

## The Japanese occupation (1942–1945) and the Indonesian war of independence

After the Japanese military occupied Java in March 1942, the Japanese military administration closed the medical schools in Batavia and Surabaya.^74^ On 29 April 1943, the birthday of the Japanese emperor, the Batavia Medical School was reopened under the name *Ika Daigaku* (Japanese: Medical College). Like Java’s medical services, the *Ika Daigaku* was run under the supervision of Japanese physicians while Indonesian physicians were in charge of everyday operations, as most European physicians had been interned. Because the Japanese had outlawed the Dutch language, medical teaching had to be conducted in Malay. To develop an appropriate medical vocabulary, Indonesian physicians established a committee to formulate Malay counterparts for Dutch and Latin medical terms on 20 October 1943.^75^ A suitable medical language would be essential for the future of the Indonesian medical profession.^76^ The Committee was placed under the auspices of the head of the Japanese Teaching Office and received assistance from several nationalist and literary figures. The task of the larger commission was to develop a standard terminology and grammar for the Indonesian language.

Until the early 1920s, Indonesian physicians had associated progress and modernity with the colonial presence of the Netherlands. In the 1920s, they came to associate these with medical science, which was inherently cosmopolitan in nature. By embracing the initiatives of the Rockefeller Foundation, they associated themselves with a competing medical and imperial power, which advocated alternative views on the organisation of medical care. During the Japanese medical occupation, Indonesian physicians benefited by associating themselves with yet another competing source of medical and imperial authority: the Japanese, which promoted an anti-Western and trans-Asian modernity, which was embodied in the Great East Asian Co-Prosperity Sphere. Japan’s views on modernity contained Western elements, which were transformed and incorporated into its own ideals. Japanese physicians provided alternate views on public health and medical care, which had originated in Germany. By aligning themselves with the Japanese, Indonesian physicians were able to emancipate themselves from the Dutch to a greater extent than ever before. After Japan’s capitulation in 1945, this alignment caused severe problems for the profession’s main representatives – as it did for several others. The position of Abdul Rasyid, for example, became untenable and he disappeared from Indonesia’s medical scene.

The Association of Indonesian Physicians (thus renamed in 1938) was disbanded – as were all organisations established in the Dutch East Indies – but continued informally under the name *Perkumpulan Tabib Indonesia* (PERTABIN; Indonesian Medical Association). Its name included the word *tabib*, which refers to traditional Islamic healers, instead of *dokter*, which refers to (Western) physicians. The association continued to be led by Abdul Rasyid, who enthusiastically endorsed Japanese medical ideas. It continued to unite most Indies physicians, who were enthralled by the role of medicine in nation building as envisaged by Japanese physicians. They had introduced them to their conceptions of public health, which were derived from German approaches but had become integrated in Japanese medical thinking.^77^ For Japanese physicians, medicine and public health were essential tools for nation building, just as it had been in the medical nationalism that had been advocated by the Association of Indies Physicians during the 1930s.^78^ In a speech to Indonesia’s physicians in 1943, future Indonesian president Sukarno emphasised that their work was not limited to ‘*treating the sick*, but also to look after and take care of the Indonesian people so that they become a very healthy and physically strong nation (*rakyat sehat dan kuat*)’.^79^ This last phrase became the main health slogan of the 1950s. The Japanese established neighbourhood associations across Indonesia as a conduit for both political propaganda and public health education; they intended to establish clinics in all of them. These neighbourhood associations were later used for the same purposes by the Suharto regime and many still operate today.

The Japanese capitulated on 15 August 1945, days after nuclear warheads exploded over Hiroshima and Nagasaki. Two days later, Sukarno and Mohamad Hatta, Indonesia’s future vice president, declared Indonesia’s independence in a modest ceremony in front of the former’s house, not far from the medical school. Three weeks later, the medical school reopened under the name Institute for Higher Medical Education (*Balai Perguruan Tinggi Kedoktoran*), and medical education commenced again.^80^ Soon after, the Dutch sent troops to reclaim their former colony. A brutal neocolonial war followed, which led to armed confrontations, deprivation, destruction, death and widespread suffering. Because the Dutch had taken control of Jakarta, the Indonesian government moved to Yogyakarta on 4 January 1946. Most academic physicians and medical students followed them to the heartland of Java, where they continued to receive medical education in various locations under improvised conditions. During the second Dutch military incursion, which commenced on 19 December 1948, the Dutch military occupied most of central Java. The Indonesian medical community joined the guerrilla forces of the Indonesian armed forces, thereby reinforcing their strong nationalistic image. Unfortunately for the Dutch, the international community turned against it and negotiations for the transfer of sovereignty commenced under the auspices of the United Nations. The actual transfer took place on 27 December 1949.

## Medicine in independent Indonesia

After the transfer of sovereignty on 27 December 1949, the political and social environment for medical science, medical education and medical care changed dramatically. The Indonesian government aimed to transform colonial institutions for higher education into institutions educating professionals to staff its bureaucracy, schools and businesses. The Indonesian Medical Association (*Ikatan Dokter Indonesia*) was founded in 1950.^81^ It did not incorporate its forerunners, the Association of Indonesian Physicians and PERTABIN, because of the close ties of the latter to the Japanese occupying forces. It had no interest in incorporating the colonial Association for the Advancement of Medical Science in the Dutch Indies or the Association of (European) Physicians either. In the 1950s, Indonesians faced the daunting task of establishing government institutions and a national infrastructure during a period of economic instability, political unrest and various insurrections. Ideas about development during the Sukarno era (1950–1965) resembled those formulated during colonial times: modernization was the main goal, experts and administrators were seen as essential actors in this endeavour, and (foreign) experts were highly valued. Sukarno diverged from these ideals by emphasising national sovereignty in matters of food and industrial production. In his aspirations, Indonesians would no longer need to rely on food imports. He also did not want Indonesia to become a country that relied on exporting its natural resources; instead, he wanted Indonesia to develop a capacity to extract and manage those natural resources itself.^82^

In 1951, the Ministry of Education and Culture established a program for higher education for independent Indonesia.^83^ It first of all highlighted the needs and demands of the state and asserted that these took precedence over those of pure research – or, as it was expressed in the Ministry’s words, research and higher education needed to develop in harmony with the state. Second, to stimulate the development of the Indonesian scientific community, universities and research institutions had to give precedence to Indonesians when hiring staff. Promising future academics received scholarships for advanced study abroad. Third, the Dutch language was outlawed in favour of Indonesian; English was tolerated, but few Indonesians spoke it. Finally, instead of graduating small numbers of highly qualified academic researchers, as had been the aim of the Batavia Medical School, Indonesian universities were instructed to produce large numbers of professionals.

To meet the country’s urgent need for more physicians, instructors associated with the Faculty of Medicine at the University of Indonesia (which was formed around the former Batavia Medical School) reformed medical education following the American model, which is also known as ‘guided study’.^84^ Medical education became cohort-based, attendance at lectures and practical classes compulsory, and examinations were held regularly to check each student’s progress. The academic model of the Batavia Medical School was abandoned in favour of a strategy that would produce the large numbers of physicians that the newly independent country needed. This strategy had been unacceptable to Indonesian physicians during colonial times because it hampered their emancipation *vis-à-vis* their European colleagues. After independence, it was seen as an utter necessity. In 1952, the University of Indonesia entered into an agreement with the University of California at San Francisco to implement a new medical curriculum. American lecturers spent several months each year in Jakarta while Indonesian medical staff travelled to the United States for advanced instruction. The number of medical graduates steadily increased in the early 1960s.^85^ Several new medical schools were established as well.

## Conclusion

In this article, I have investigated the growth of medical education in the Dutch East Indies and Indonesia and the strong adherence of the Indonesian medical community to ideals of progress and modernity. I have analysed the creation of a culturally hybrid Indonesian medical profession, which closely resembled the European group and therefore occupied an ambiguous place in colonial society. Medical education in the Dutch East Indies had a modest start. In 1851, the colonial administration of the Dutch East Indies established a two-year medical course for indigenous students at the military hospital in the colony’s capital, Batavia (today’s Jakarta). It was named the *Dokter Djawa School* (School for Javanese Physicians) and aimed to educate medical assistants and vaccinators. During the following 75 years, the medical curriculum was continuously improved, expanded and transformed. From the 1880s on, when future Nobel laureate Christiaan Eijkman became director of the Batavia medical college, the education it provided used the latest medical technologies available and taught the most recent medical insights. Training was again upgraded in 1913, as graduates from the Batavia medical college received the degree of *Inlandsch Arts* after following an extensive course of study lasting nine years (three years preparatory education and a six-year medical course). The school was renamed School for the Education of Indies Physicians (*School tot Opleiding van Indische Artsen;* the acronym remained STOVIA). The same year, a second medical college following the same curriculum as its counterpart in Batavia was established in Surabaya; it was named the Netherlands Indies School for Physicians (*Netherlandsch-Indische Artsen School*; NIAS). Both schools offered practical or professional rather than academic medical degrees. After the bacteriological and parasitological revolutions in medicine, and after the entry of X-ray technology in the Indies, the esteem of modern, Western medicine rose tremendously, which promised successful career paths for the graduates of the medical colleges in the Indies. In 1927, a medical school offering a course of study equivalent to that offered at Dutch universities commenced operations in Batavia, named the School for Higher Medical Education (*Geneeskundige Hoogeschool*; GH). In the following years, the STOVIA gradually suspended operations while the NIAS continued operating with its curriculum unchanged.

Indies physicians were disliked by their European colleagues when they aspired to be more than assistants. In addition, the Indonesian medical community often featured in public debates on the nature and desirability of the Ethical Policy, the Dutch civilising mission for the colonies which was proclaimed in 1901. For colonial progressives, Indies physicians were educated Indonesians who, at some undefined point in the future, could take responsibility for medical care in the Indies. They indicated the success of their modernising mission. Opponents of the Ethical Policy depicted them as rootless and alienated urban-based imitation Europeans who could only be expected to foment unrest and social upheaval.

The Indonesian medical community itself was initially motivated by a desire for emancipation and equal conditions with their European colleagues. Emphasising that their professional predicament was inherently associated with the nature of colonial society, they analysed the nature of the colonial social body and its ailments. They became involved in the Indonesian nationalist movement and were active as journalists, publishers, organisers and politicians, thereby becoming public intellectuals. Even though Indies physicians became well known for their social and political engagement, it was another development that ultimately shaped the medical profession after World War II. Through scholarships for advanced medical study in the Netherlands and through the establishment of the Batavia Medical School in 1927, a small elite corps of Indonesian physicians came into being. These elite physicians were generally not interested in politics but focused on creating a viable Indonesian medical profession. It was this group that shaped medical care, medical education and public health initiatives in independent Indonesia.

## Footnotes

1 The effects of receiving a medical education have been investigated by medical sociologists who focused on investigating students at elite American medical schools. For a few classical studies see: Robert K. Merton , George G. Reader and Patricia L. Kendall , The Student-Physician: Introductory Studies in the Sociology of Medical Education (Cambridge: Harvard University Press for the Commonwealth Fund, 1957); Howard S. Becker , Blanche Geer , Everett C. Hughes , and Anselm M. Strauss , Boys in White: Student Culture in Medical School (Chicago: University of Chicago Press, 1961); Renée C. Fox , Experiment Perilous: Physicians and Patients Facing the Unknown (New Brunswick, NJ: Transaction, 1998); and Renée C. Fox , Essays in Medical Sociology: Journeys into the Field (New Brunswick, NJ: Transaction, 1988). Thus far, little research has been conducted on non-Western medical students, but see Claire Wendland , A Heart for the Work: Journeys through an African Medical School (Chicago: University of Chicago Press, 2010).

2 Two contributions stand out: A. de Waart, ‘Vijf-en-Zeventig Jaren Medisch Onderwijs te Weltevreden [75 Years Medical Education in Central Batavia]’, in A. de Waart (ed.), *Ontwikkeling van het Geneeskundig Onderwijs te Weltevreden, 1851–1926* (Weltevreden: Kolff, 1926) and M. A. Hanafia, Bahder Djohan and Surono, *125 Tahun Pendidikan Dokter di Indonesia 1851–1976* [125 Years of Medical Education in Indonesia] (Jakarta: FKUI, 1976).

3 See, for example, Daniël de Moulin , ‘Teaching of Medicine in the Dutch East Indies’, Clio Medica, 21, 1–4 (1987/1988), 119–242475292.

4

Liesbeth Hesselink , Healers on the Colonial Market: Native Doctors and Midwives in the Dutch East Indies (Leiden: KITLV Press, 2011).

5

Hans Pols , Nurturing Indonesia: Medicine and Decolonisation in the Dutch East Indies (Cambridge: Cambridge University Press, 2018).

6 Until 1911, the Dutch East Indies only had a military medical service, which aimed to maintain the health of the colonial armed forces as the necessary backbone of the Dutch colonial enterprise. It also served the European inhabitants of the Indies when there was spare capacity. Until the turn of the twentieth century, the colonial administration of the Dutch East Indies did not count the medical care of the indigenous population as its responsibility, except in the case that indigenous people were under its immediate responsibility (as prisoners, forced labourers and the insane) or in the case of epidemics.

7 On the extensive exchange of letters and the many disagreements between Willem Bosch, governor-general J. J. Rochussen and Minister of Colonies J. C. Baud see Hesselink, *op cit.* (note 4), 60–73. For the history of medical education in the Dutch East Indies, see also S. Somadikarta , Tri Wahyuning M. Irsyam and Boen S. Oemarjati , Tahun Emas Universitas Indonesia, 3 vols. (Jakarta: Universitas Idonesia Press, 2000). For a history of the Surabaya medical college and the Airlangga University’s Faculty of Medicine, see: Sandiantoro, *Jejak Jiwa di Lintasan Zaman (1913–2013): Menganang yang Silam, Meretas Masa Depan: Peringatan Satu Abad Pendidikan Dokter di Surabaya* [One Century of Medical Education in Surabaya] (Surabaya: Panitia Peringatan Satu Abad Pendidikan Dokter di Surabaya, 2013).

8 This college was inspired by French initiatives in this domain. See Daniël de Moulin,*‘s-Rijksweekschool voor Militaire Geneeskundigen te Utrecht (1822–1865)* [The Training College for Medical Officers at Utrecht (1822–1865)] (Amsterdam: Rodopi, 1987), in particular Annemarie de Knecht-van Eekelen, ‘De Invloed van ’s-Rijkskweekschool voor Militaire Geneeskundigen op de Geneeskunde in Nederlandsch Oost-Indië [The Influence of the State School for Medical Officers on Medicine in the Dutch East Indies]’, in Daniël de Moulin (ed.),*‘s-Rijksweekschool voor Militaire Geneeskundigen te Utrecht (1822–1865)* (Amsterdam: Rodopi, 1987), 94–109.

9 For the early history of the *Dokter Djawa School,* see Hesselink, *op cit. (note 4)*, 75–118.

10 Hesselink, *ibid.*, 75–81.

11 J. Alken, ‘De School voor Doctor Djawa [The Doctor Djawa School]’, *Indisch Genootschap* (28 February 1882), 2.

12 J. A. Fles, *Kort Antwoord op het ‘Nog Eens: De Kweekschool voor Militaire Geneeskundigen’, van den Hoogleeraar F. C. Donders* [Short Response to ‘Again: The Training School for Medical Officers’, by Professor F.C. Donders] (Utrecht: Dekema, 1867), 6.

13 T. G. van Vogelpoel, ‘Het Onderwijs in de Anatomie [The Education in Anatomy]’, in A. de Waart (ed.), *Ontwikkeling van het Geneeskundig Onderwijs te Weltevreden, 1851–1926* (Weltevreden: Kolff, 1926), 143.

14 Vogelpoel, *ibid.*, 145.

15 Vogelpoel, *ibid.*, 149.

16 A. E. Sitsen, ‘Verslag over 173 Lijkopeningen, Verricht aan de Stovia te Weltevreden [Report over 173 Dissections Conducted at the Stovia]’, *Geneeskundig Tijdschrift voor Nederlands Indië*, 54 (1914), 191–203.

17 K. Groeneboer, *Weg tot het Westen: Het Nederlands voor Indië 1600–1950: Een Taalpolitieke Geschiedenis* [Avenue to the West: The Dutch Langauge in the Dutch East Indies] (Leiden: KITLV Press, 1993). For indigenous attitudes towards development and progress and the Dutch language, see Ahmat B. Adam , The Vernacular Press and the Emergence of Modern Indonesian Consciousness (Ithaca, NY: Southeast Asia Program, Cornell University, 1995).

18 T. A. Kandou, ‘De Minahassers en ‘t Nederlands [The People from the Minahassa and the Dutch Language]’, *Orgaan van de Studeerenden-Vereeniging Minahassa,* 1, 2 (15 March 1918), 28.

19 For a brief biography, see Krzysztof Pietrzak , ‘Christiaan Eijkman (1856–1930)’, Journal of Neurology, 266, 11 (Nov 2019), 2893–530588543 10.1007/s00415-018-9162-7PMC6803585.

20

Kenneth J. Carpenter , Beriberi, White Rice, and Vitamin B: A Disease, a Cause, and a Cure (Berkeley: University of California Press, 2000).

21 De Waart, *op. cit.* (note 2), 17, 18.

22

Laurence Monnais and Hans Pols , ‘Health and Disease in the Colonies: Medicine in the Age of Empire’, in Robert Aldrich and Kirsten McKenzie (eds.), The Routledge History of Western Empires (New York: Routledge, 2014), 270–84.

23 For the results, see W. Schüffner and W. A. Kuenen, *De Gezondheidstoestand der Arbeiders, Verbonden aan de Senembah-Maatschappij op Sumatra, Gedurende de Jaren 1897 tot 1907* [The State of Health of Workers Associated with the Senembah Planation on Sumatra 1897–1907] (Amsterdam: de Bussy, 1910). See also C. W. Janssen, *Senembah Maatschappij, 1889–1914* [Senembah Company, 1889–1914] (Amsterdam: Roeloffzen, 1914) and Hans Pols , ‘Quarantine in the Dutch East Indies’, in Alison Bashford (ed.), Quarantine: Local and Global Histories (Basingstoke: Palgrave Macmillan, 2016), 85–102 .

24

Erich Brenner , ‘Human Body Preservation: Old and New Techniques’, Journal of Anatomy, 244, 3 (2016), 316–4410.1111/joa.12160PMC393154424438435.

25 H. F. Roll, *Jaarlijksch Verslag der School tot Opleiding van Inl. Artsen te Weltevreden over den Cursus 1904* [Annual Report Stovia 1904] (Batavia: Landsdrukkerij, 1904), 18–19; Vogelpoel, *op. cit.* (note 13), 152.

26 For the Ethical Policy, see Elsbeth Locher-Scholten, *Ethiek in Fragmenten: Vijf Studies over Koloniaal Denken en Doen van Nederlanders in de Indonesische Archipel, 1877–1942* [Ethics in Fragments] (Utrecht: H&S, 1981); Robert Cribb , ‘Development Policy in the Early 20^th^ Century’, in Jan-Paul Dirkse , Frans Hüsken and Mario Rutten (eds.), Development and Social Welfare: Indonesia’s Experiences under the New Order (Leiden: KITLV Press, 1993), 225–45; Suzanne Moon , Technology and Technical Idealism: A History of Development in the Netherlands East Indies (Leiden: CNWS, 2007).

27 The involvement of the Rockefeller Foundation on international health and medicine, including medical education, has been extensive. See, for example, Randall Packard , A History of Global Health: Interventions into the Lives of Other Peoples (Baltimore, MD: Johns Hopkins University Press, 2014); John Farley , To Cast Out Disease: A History of the International Health Division of the Rockefeller Foundation (1913–1951) (New York: Oxford University Press, 2004); Liew Kai Khiun , ‘Wats and Worms: The Activities of the Rockefeller Foundation’s International Health Board in Southeast Asia (1913–1940)’, in Laurence Monnais and Harold J. Cook (eds.) Global Movements, Local Concerns: Medicine and Health in Southeast Asia (Singapore: Singapore University Press, 2012), 43–61 . The Rockefeller Foundation was active in the Dutch East Indies in public health demonstration projects in the Banyumas area. See also Hohee Cho’s contribution in this issue.

28 Victor Heiser’s Diary, Notes on the 1916 trip, vol. 1, page 248. Rockefeller Archive Center, Rockefeller Foundation Records, Officer’s diaries, Record Group 12. For Heiser, see also Tomoko Akami , ‘Imperial Polities, Intercolonialism, and the Shaping of Global Governing Norms: Public Health Expert Networks in Asia and the League of Nations Health Organization, 1908–37’, Journal of Global History, 12, 1 (2017), 4–25 .

29 W. S. Carter, Diary, Entry for Tuesday, March 20, 1934. Rockefeller Archives Centre, Rockefeller Foundation Records, Record Group/Series 1.1 Projects: 655 JAVA. Box 1, folder 1.

30 W. S. Carter, Medical Education in Java: Report of a Survey Made March 16^th^ – April 4^th^, 1924. Rockefeller Archives, Rockefeller Foundation Records, Record Group/Series 1.1. Project 655: Java. Box 1, folder 3.

31 Carter, *ibid.*, 108.

32 On the social background of the medical students in the Indies, see Pols, *op. cit.* (note 5), 47–9.

33 For an analysis of the different layers of the indigenous aristocracy, see Heather Sutherland , The Making of a Bureaucratic Elite: The Colonial Transformation of the Javanese Priyayi (Singapore: Heinemann Asia, 1979).

34 This phrase was coined by C. D. de Langen, instructor in internal medicine at the STOVIA and, later, at the Batavia Medical School. See C. D. de Langen, ‘Beroepsbelangen [Professional Interests]’, *Orgaan der Vereeniging van Indische Geneeskundigen*, 16, 1 (1927), 13.

35 For more on ideals of progress and development in Indonesian medicine, see Hans Pols , ‘Decolonising Science and Medicine in Indonesia’, in Andrew Goss (ed.), The Routledge Handbook of Science and Empire (New York: Routledge, 2021), 300–13.

36 Adam, *op. cit.* (note 17).

37

David Scott , Conscripts of Modernity: The Tragedy of Colonial Enlightenment (Durham, NC: Duke University Press, 2004). For cosmopolitanism, see Anthony Appiah , Cosmopolitanism: Ethics in a World of Strangers (New York: Norton, 2006).

38 For the school almanacs of the STOVIA, see, for example, Bahder Djohan, A. Ramali, A. Gafar, R. Mochtar, Soekandar, M.A. Hanafiah, Zainoedin, Loekmono, and Darwis (eds.), *STOVIA-Almanak voor het Jaar Negentien Honderd Vier en Twintig* [STOVIA Yearbook 1924] (Weltevreden: Kolff, 1924); Bahder Djohan, Ahmad Ramali, Abdoel Gafar, Soekendar, Darwis, Loekmono, and Zainoedin (eds.), *STOVIA-Almanak voor het Jaar Negentien Honderd Zes en Twintig* [STOVIA Yearbook 1926] (Weltevreden: Kolff, 1926). The students at the NIAS published a newspaper entitled *NIAS Orgaan* and several school almanacs as well, such as Moersita, Soehardo, S. Rachmat, Sosodoro, K.A. Tan, Mohamad, and Djafar Siregar (eds.), *Jaarboek 1932 Uitgegeven door de NIAS Vereeniging te Soerabaja* (Surabaya: Kolff, 1932) And Dj. Siregar, Soeharso and C. Schreuder (eds.), *NIAS Almanak, Lustrumnummer 1933–1934* (Batavia: Kolff, 1934).

39 See Taufik Abdullah , Indonesia: Towards Democracy (Singapore: ISEAS, 2009); Akira Nagazumi , The Dawn of Indonesian Nationalism: The Early Years of the Budi Utomo, 1908–1918 (Tokyo: Institute of Developing Economies, 1972). See also Bambang Eryudhawan (ed.), 100 Tahun Kebangkitan Nasional: Jejak Boedi Oetomo, Peristiwa, Tokoh dan Tempat [One hundred years of national awakening] (Jakarta: Badan Pelestarian Pusaka Indonesia (BPPI), 2009).

40 This phrase was first used by one of the most outspoken advocates of the Ethical Policy. See C. T. van Deventer, ‘Insulinde’s Toekomst [Insulinde’s Future]’, *De Gids,* 72, 3 (July 1908), 69–99.

41 The term ‘imagined communities’ is of course derived from Benedict Anderson’s influential work, which was partly based on his previous studies on the Dutch East Indies. Educational facilities and the Indies youth movements play an important role in his narrative. See Benedict Anderson , Imagined Communities: Reflections on the Origin and Spread of Nationalism, Revised and extended edn. (London: Verso, 2006 [1983]).

42 Kees Mandemakers, *HBS en Gymnasium: Ontwikkeling, Structuur, Sociale Achtergrond en Schoolprestaties, Nederland, circa 1800–1968* [HBS and the Gymnasium: Development, Structure, Social Background, and Results] (Amsterdam: IISG, 1996).

43 Willem Daniëls, *De MULO: De Carrièreschool voor het ‘Gewone’ Volk* [The MULO: The School for Career-Minded Common Folk] (Amsterdam: Prometheus, 2018).

44 H. W. van den Doel, ‘Indianisatie en Ambtenarensalarissen [Indianisation and the Salaries of Civil Servants]’, *Tijdschrift voor Geschiedenis*, 100, 4 (1987), 556–79.

45

Ann Laura Stoler , Carnal Knowledge and Imperial Power: Race and the Intimate in Colonial Rule (Berkeley: University of California Press, 2002), 79 .

46 Pols, *op cit.* (note 5), 100–5; *Jubileumnummer 1911–1936: Orgaan Vereeniging van Indische Geneeskundigen* [Anniversary Volume 1911–1936: Organ of the Association of Indies Physicians], (Batavia: G. Kolff, 1937).

47 Pols, *op. cit.* (note 5), 107–14.

48 Pols, *ibid.*, 138–60. For the Rockefeller initiatives see: J. L. Hydrick , Intensive Rural Hygiene Work and Public Health Education of the Public Health Service of Netherlands India (Batavia: Author, 1937).

49 On Rivai and several of his colleagues who employed similar initiatives see Pols, *op. cit.* (note 5), 21–45.

50 The first book-length critique of the Ethical Policy was formulated by conservative physician J. H. F. Kohlbrugge in his *Blikken in het Zieleleven van den Javaan en Zijner Overheerschers* [Glances into the Soul of the Javanese and His Rulers] (Leiden: Brill, 1907), which took aim at the STOVIA several times.

51 J. Bijker, *Rapport der Commissie tot Voorbereiding eener Reorganisatie van den Burgerlijken Geneeskundigen Dienst* [Report of the Commission for the Preparation of a Reorganisation of the Civil Health Service] (Batavia: Landsdrukkerij, 1908). For this report and the ensuing discussions, see Pols, *op cit.* (note 5), 97–100.

52 Bijker, *op cit.* (note 51), 72; 70.

53 H. F. Roll, *Is Reorganisatie van de School tot Opleiding van Inlandsche Artsen te Weltevreden Nogmaals Nodig?* [Is a Reorganization of Medical Instruction of Native Physicians in Weltevreden Again Necessary?] (Dordrecht: Morks & Geuze, 1909).

54 W. K. Tehupeiory, ‘Iets over de Inlandsche Geneeskundigen [About Native Physicians]’, *Indisch Genootschap: Verslagen der Vergaderingen* (28 January 1908), 101–34.

55 H. J. D. Apituley, R. Tumbelaka, H. F. Lumentut, Radjiman, M. Brenthel Moh. Salih, Ph. Laoh, Abdul Rivai, *Eenige Opmerkingen naar aanleiding van de Voorstellen tot Reorganisatie van den Civiel Geneeskundigen Dienst in Nederl. Oost-Indië* [Comments on the Propsoals for the Reorganization of the Civil Medical Service in the Dutch East Indies] (Amsterdam: Eisendrath, 1910).

56 Hoofdbestuur [Main Board], ‘Het Indisch Ontwerp 1913 [The Indies Proposal 1913]’, *Bond van Geneesheeren in N.-I.*, 52-53 (1912), 29. See also Pols, *op. cit.* (note 5), 105–7.

57 The name of this institution is complicated because it offered medical education at the level of Dutch university-affiliated medical schools in the absence of a university in the Dutch East Indies, hence School for Higher Medical Education.

58 W. S. Carter Diary, Tuesday, 20 March 1934. Reporting on an interview with Professor Cornelis Bonne. Rockefeller Archives, Rockefeller Foundation Records, Record Group/Series 1.1. Project 655: Java. Box 1, file 1.

59 Report 655 L on Public Health Education in Java, 1928–1929, note dated 8 August 1928. Rockefeller Archive Centre, Rockefeller Foundation Records, Record Group 1.1., Projects. Series 655: Java, box 2, file 1.

60 W. S. Carter, Report of March 1928, on the University Faculty of Medicine, Batavia, Java, 162. Rockefeller Archives, Rockefeller Foundation Records, Record Group/Series 1.1. Project 655: Java. Box 1, file 2.

61 Walch’s oration was published as: E. W. Walch, *Het Goed Recht der Hygiëne, *hare* Toepassing in Ned. Indië: Inaugurele Rede [The Prerogative of Hygiene: Its Application in the Dutch Indies]* (Weltevreden: Kolff, 1930).

62 Preliminary results were published as Johan W. Tesch, *The Hygiene Study Ward Centre at Batavia: Planning and Preliminary Results, 1937–1941* (Leiden: Universitaire Pers, 1947) and J. E. Dinger , R. Soekonto , and J. W. Tesch , ‘Investigation into the Epidemiology of Diphtheria in the Hygiene Study Ward at Batavia’, Documenta Neerlandica et Indonesica de Morbis Tropicis, 1, 1 (1949): 3–33 18151871 . The project was continued after World War II by Raden Mochtar, then professor of public health at the faculty of medicine at the University of Indonesia.

63 For a report, see Hydrick, *op cit*. (note 48). See also Eric Andrew Stein , ‘Colonial Theatres of Proof: Representation and Laughter in 1930s Rockefeller Foundation Hygiene Cinema in Java’, Health and History, 8, 2 (2006), 14–44 .

64 Just before, the *Uijver* had received wide acclaim by winning the London to Melbourne race. See, for example, ‘De Ontzettende Ramp van den “Uijver”: Het Toestel met de Inzittenden Totaal Verbrand’ [The Terrible Disaster of the “Uijver”: The Airplane with All Passengers Totally Burnt Down], *Het Nieuws van den Dag voor Nederlandsch-Indië* 22 December 1934, 1.

65

Budi Agustono , Junaidi and Kiki Maulana Affandi , ‘Pathology Laboratory: An Institution of Tropical Diseases in Medan, East Sumatra, 1906–1942’, Cogent Arts & Humanities, 8, 1 (2021), 1905261; 10.1080/23311983 ; Jan Peter Verhave , The Moses of Malaria: Nicolaas H. Swellengrebel (1885–1970) Abroad and at Home (Rotterdam: Erasmus, 2011).

66 Several of his colleagues at the Pathological Laboratory had similar career trajectories. Among them were Willem A. Kuenen, Johannes J. van Lochem, Nicolaas H. Swellengrebel, E. P. Snijders and Willem Kouwenaar, who all were appointed by the University of Amsterdam and worked at the Institute of Tropical Hygiene at the Royal Institute of the Tropics.

67 They include Sjoeib Proehoeman, Zainal and Achmad Mochtar.

68 For De Vogel’s blueprint of the Public Health Service, see W. T. Vogel, *De Taak van den Burgelijken Geneeskundigen Dienst in Nederlandsch-Indië* [The Task of the Civil Health Service in the Dutch Indies] (Amsterdam: Koloniaal Instituut, 1917).

69 W. T. de Vogel, ‘Anophelines dans l’eau de Mer’, *Società per gli studi della Malaria,* 8 (1907), 1–18. See also Andrew Balfour , ‘Musquito Breeding in Saline Waters’, Bulletin of Entomological Research, 12, 1 (1921), 29–34 , which calls De Vogel’s contribution as ‘amongst the more important of the earlier investigations’ (p. 30).

70 Best known are the anti-malaria initiatives in Siboga; see W. T. de Vogel , ‘Report of the Investigations Carried Out with Regard to the Sanitary Condition of the Port Sibolga’, Mededeelingen van den Burgerlijken Geneeskundigen Dienst in Nederlandsch Indië, 4 (1915).

71 Pols, *op cit.* (note 5), 139–41.

72 A similar change in political orientation can be found among Taiwanese physicians when Taiwan was under Japanese control. When they were considered second-rate physicians, Taiwanese doctors portrayed themselves as ‘physicians of the nation’ and were active in social and political movements. In the 1930s, they were treated as colleagues, upon which their political activism greatly diminished. See Ming-Cheng M. Lo , Doctors within Borders: Profession, Ethnicity, and Modernity in Colonial Taiwan (Berkeley, CA: University of California Press, 2002).

73 Pols, *op. cit.* (note 5), 133–5.

74 For developments during the Japanese occupation, see Pols, *op. cit.* (note 5), 161–82.

75 Achmad Ramali was associated with this committee. The results were published as Ahmad Ramali and K. St. Pamoentjak, *Kamus Kedoktoran: Arti dan Keterangan Istilah* [Medical Dictionary] (Djakarta: Djambatan, 1953). The most recent edition is: Ahmad Ramali, K. St. Pamoentjak and Hendra T. Laksman, *Kamus Kedoktoran: Arti dan Keterangan Istilah* [Medical Dictionary], 25^th^ edn. (Djakarta: Djambatan, 2003).

76 Mardanas Safwan, *Prof. Dr. Bahder Djohan: Karya dan Pengabdiannya* [Prof. Dr. Bahder Djohan] (Jakarta: Proyek Inventarisasi dan Dokumentasi Sejarah Nasional (Indonesia), 1985), 21.

77

Hoi-eun Kim , Doctors of Empire: Medical and Cultural Encounters between Imperial Germany and Meiji Japan (Toronto: University of Toronto Press, 2014).

78 Pols, *op. cit.* (note 5), 161–82.

79 Quoted in Pols, *ibid.*, 167.

80 For developments during the Indonesian war of independence, see Pols, *op. cit.* (note 5), 183–203.

81 ‘Announcement [On the Founding IDI]’, *OSR News,* 2, 11 (1950), 141–2.

82 For an overview of Sukarno’s political ideas, see Bradley R. Simpson , Economists with Guns: Authoritarian Development and US–Indonesian Relations, 1960–1968 (Stanford, CA: Stanford University Press, 2008).

83 See R. Thomas Murray , A Chronicle of Indonesian Higher Education: The First Half Century, 1920–1970 (Singapore: Chopmen Enterprises, 1973), 40–172 ; William K. Cummings and Salman Kasenda , ‘The Origins of Modern Indonesian Higher Education’, in Philip G. Altbach and Viswanathan Selvaratnam (eds.), From Dependence to Autonomy: The Development of Asian Universities (Dordrecht: Kluwer, 1989), 143–66; R. M. Koentjaraningrat and Harsja W. Bachtiar , ‘Higher Education in the Social Sciences in Indonesia’, in R. M. Koentjaraningrat (ed.), The Social Sciences in Indonesia (Jakarta: LIPI, 1975), 1–42 ; Bachtiar Rifai and Koesnadi Hardjasoemantri , Perguruan Tinggi di Indonesia [Higher Education in Indonesia] (Jakarta: Departemen Perguruan Tinggi dan Ilmu Pengetahuan, 1965).

84

Vivek Neelakantan , Science, Public Health and Nation-Building in Soekarno-Era Indonesia (Newcastle upon Tyne: Cambridge Scholars, 2017), 142–55. See also Francis Scott Smyth , ‘University of California Medical Science Teaching in Indonesia’, Journal of Medical Education, 32, 5 (1957), 344–4913416838 10.1097/00001888-195705000-00006; Francis Scott Smyth , ‘Health and Medicine in Indonesia’, Journal of Medical Education, 38, 8 (1963), 693–9613989653 ; John S. Wellington , ‘Medical Science and Technology’, in Howard M. Beers (ed.), Indonesia: Resource and Their Technological Development (Lexington, KT: University Press of Kentucky, 1970), 165–73; Willard A. Hanna , ‘A Bi-national Project in Medical Education’, American Universities Field Staff, Southeast Asia Series, 4, 19 (1956), 1–10 .

85 During the 1950s, there was an extreme shortage of physicians in Indonesia. In 1957, for example, there were 1 450 physicians working there, which is roughly equal to one physician for every 55 000 persons. See Willard A. Hanna , ‘No Place for Hypochondriacs: Medical Services in Indonesia’, American Universities Field Staff Reports, Southeast Asia Series, 5, 5 (1957), 1–5 .

